# Using Intervention Mapping to Develop Health Education Components to Increase Colorectal Cancer Screening in Puerto Rico

**DOI:** 10.3389/fpubh.2017.00324

**Published:** 2017-12-07

**Authors:** Yolanda A. Serra, Vivian Colón-López, Lara S. Savas, Sally W. Vernon, Natalie Fernández-Espada, Camille Vélez, Alelí Ayala, María E. Fernández

**Affiliations:** ^1^The University of Texas Health Science Center at Houston, School of Public Health, Houston, TX, United States; ^2^Cancer Control and Population Sciences Program, University of Puerto Rico Comprehensive Cancer Center, San Juan, Puerto Rico; ^3^UPR-MDACC Partnership for Excellence in Cancer Research, Medical Sciences Campus, University of Puerto Rico, San Juan, Puerto Rico

**Keywords:** intervention mapping, colorectal cancer screening, entertainment education, behavioral journalism, self-efficacy, program development, behavioral change, Federally Qualified Health Centers

## Abstract

**Introduction:**

Colorectal cancer (CRC) is a leading cause of cancer-related mortality in Puerto Rico (PR). Although largely preventable through screening and treatment of precancerous polyps, CRC screening rates in PR remain low while CRC incidence and mortality continue to increase.

**Methods:**

We used intervention mapping (IM), a systematic framework using theory and evidence to plan a health promotion intervention to increase colorectal cancer screening (CRCS) among Puerto Rican adults 50 years and older who are patients of Federally Qualified Health Centers (FQHCs) in PR.

**Results:**

To inform the development of a logic model of the problem during the needs assessment phase, we determined the CRC incidence and mortality rates in PR using recent data from the PR Cancer Registry, conducted a literature review to better understand behavioral and environmental factors influencing CRC among Hispanics in general and in Puerto Ricans, and collected new data. We conducted seven focus groups to identify community needs and resources, specific sub-behaviors related to CRCS (performance objectives) and the determinants of CRCS. We then developed matrices of change objectives that would guide the content, behavioral change method selection, and the practical applications that would be included in the program. We selected two overarching methods: entertainment education and behavioral journalism and developed practical applications, materials, and messages containing several other methods including modeling, persuasion, information, and tailoring. We developed and pretested a Tailored Interactive Multimedia Intervention, newsletter, an action plan, and supplemental print materials for patients. We also developed a patient mediated provider prompt to increase provider recommendation and improve patient provider communication.

**Conclusion:**

The use of IM for systematic planning produced a detailed coherent plan for the CRCS educational intervention. Guided by IM processes, steps, and tasks, we used community level information, existing literature, theory, and new data to develop health education materials that were well received by the priority population and will likely increase CRCS among FQHC patients in PR.

## Introduction

In Puerto Rico (PR), colorectal cancer (CRC) is the second leading cause of death due to cancer among both men and women (1). Between 2010 and 2014, the age-adjusted incidence rate of CRC on the island was 31.3 per 100,000 population among women, and 48.0 among men (1). Colorectal cancer screening (CRCS) can reduce both, the incidence and mortality of CRC through early detection and removal of precancerous lesions (2). Currently, in PR, three types of CRCS tests are performed: fecal immunochemical test (FIT), fecal occult blood test (FOBT), and colonoscopy. The American Cancer Society and the US Preventive Task Force recommend regular CRCS between the ages of 50–75. In PR, Federally Qualified Health Centers (FQHCs) use either FIT or FOBT for CRCS. Patients with a family history of CRC and those who show risk factors are strongly recommended to have a colonoscopy, in accordance to the American Cancer Society guidelines (3). Nonetheless, the Behavioral Risk Factors Surveillance System reported that for the population over 50 years of age in PR, in 2014 only 18.5% had ever had either a FIT or FOBT and only 50.6% had ever had a sigmoidoscopy or colonoscopy (4). These screening rates are substantially lower than the goal targeted by the National Colorectal Cancer Roundtable (5), which aims to achieve 80% screening rate by 2018.

Both personal determinants such as low knowledge, fear of detection, and low perceived risk; and environmental factors such as lack of doctor’s recommendation, lack of health insurance, and issues related to the local Health-Care System (i.e., high turnover rate among providers, low number of gastroenterologists, and increased patient ratio for primary care physicians) negatively influence CRCS in PR (6–10). Another organizational barrier that affects CRCS uptake is the fact that many clinics do not have an on-site laboratory and patients are required to visit an offsite laboratory to obtain and return FIT/FOBT kits; thus complicating the screening process (6).

Salient personal determinants that negatively affect CRCS among Hispanics in the U.S. and in PR include the following: lack of knowledge and misconceptions regarding CRC and CRCS, low health literacy, social norms and negative attitudes toward screening, low perceived risk of CRC, and perceived barriers such as a lack of time, perceived high cost of testing, or difficulties with transportation (6, 8, 11–17).

The Guide to Community Preventive Services (Community Guide) recommends one-on-one education in combination with small media and patient/provider reminders as strategies to increase CRCS (18). Although evidence suggests that these strategies are effective for fecal occult blood testing (FIT/FOBT), evidence regarding these approaches for increasing colonoscopy is still insufficient (19). Currently, there are few studies examining one-on-one education in combination with other communication strategies in the Latino population (12, 16, 20) and none with Puerto Ricans. The Puerto Rico Community Cancer Control Outreach Program designed a study to address this gap by developing and evaluating a health promotion program (*Salud por la Vida*) to increase CRCS in PR. The purpose of this article is to describe how we used intervention mapping (IM), a systematic planning framework, to guide the development of the program. This effort was carried out in collaboration with the PR Colorectal Cancer Coalition and the PR Primary Health Association, among others, using principles of community based participatory research. The research protocol and the data-gathering instruments were approved by the Institutional Review Boards of the UPR-MSC and the University of Texas Health Science Center at Houston. All study participants provided informed consent prior to enrollment to the study.

## Methods

### Intervention Mapping

Intervention mapping is a systematic approach for the development of theory and evidence-based health promotion interventions and for planning their implementation (21). IM has been successfully employed to promote and increase screening practices for several types of cancers such cervical and breast cancer (22–27) and CRC (12, 28).

The IM process is composed of six steps; each one involves specific tasks (21) that guide the translation of relevant personal determinants and environmental factors into a health promotion program (29). We describe the first four steps (needs assessment, matrix of change objectives, selection of theory-based methods and practical strategies, and program production).

In step 1, we conducted a needs assessment based on the PRECEDE/PROCEED model (30) to identify the factors the program should address. In step 2, we identified the overall behavioral goal for the program and developed a matrix that combined the health-promoting behaviors and their determinants to create change objectives. During step 3, we paired change objectives with theoretical models and strategies to affect the selected determinants. Finally, we developed and pretested the program in step 4. Throughout the planning process, we used information obtained from the literature, and guided by behavioral theory, and new data to identify personal determinants and environmental factors influencing CRCS and to select the most appropriate methods and strategies to influence these (see Table 1).

**Table 1:** Intervention mapping (IM) to develop health educational components to increase colorectal cancer screening in Puerto Rico (PR) (steps 1–4).

Step 1: Logic model of the problem	Establish a planning group
	Conduct a needs assessment based on the PRECEDE/PROCEED model:
	Data from PR Registry Review of empirical and theoretical literature Focus Group
	Identify the factors the program should address

Step 2: Program outcome and matrix of change objectives	Identify overall behavior goal Specify health-promoting behaviors (performance objectives) Select determinants for behavioral outcomes Construct matrix of change objectives

Step 3: Educational components design	Identify theoretical and evidence-based change methods:
	Entertainment education Behavioral journalism Patient activation method
	Select practical applications and strategies to operationalize the methods

Step 4: Educational components production	Design format, themes, and messages of the educational components:
	Tailored Interactive Multimedia Intervention Printed materials (newsletter, infographics, and action plan) Provider prompt Patient reminder support call
	Create drafts of the educational components:
	In Spanish Adapted to the Puerto Rican culture Low health literacy
	Pretest the educational components Produce the final educational components

*This table is informed by IM steps (31)*.

### Theoretical Underpinnings

To identify factors associated with the risk behavior and with the health-promoting behavior (CRCS in this case), IM suggests an integrated and iterative approach using theory and evidence. We used constructs from the Integrated Behavioral Model (32) which includes constructs from the most commonly used theoretical models in health promotion (i.e., Social Cognitive Theory, Theory of Reasoned Action, Theory of Planned Behavior, and the Health Belief Model). These constructs are as follows: severity, susceptibility, self-efficacy, attitudes, outcome expectations, perceived barriers and facilitators, and normative believes. Consideration of these constructs provided guidance for the identification and understanding of determinants related to CRCS and the selection of theory-based methods to achieve behavioral change.

## Results

### IM Step 1: Needs Assessment

Data from the PR Registry (17) demonstrated that not only was there treatment delay for people with government health care as compared to those with other insurance coverage but survival was also lower. In addition, CRCS rates are lowest among individuals who had either no insurance or government health insurance, compared to those with other forms of insurance. Therefore, we focused on reaching these patients through a collaboration with FQHCs since their patients are primarily either uninsured or have government health insurance.

We completed an extensive review of empirical and theoretical literature to identify factors influencing CRCS in US Hispanic populations and PR. As mention earlier, we used the Integrated Model to guide exploration of potential constructs (determinants). We also identified determinants that were either correlates or predictors of CRCS among Hispanics from empirical studies. For example, a systematic review (33) assessed theory-based constructs associated with CRCS. These constructs included perceived susceptibility and severity, benefits and barriers, and self-efficacy from the health belief model (34, 35); attitudes, social norms and perceived behavior control from the theory of reasoned action and the theory of planned behavior (36, 37); self-efficacy from social cognitive theory (38); and decisional balance from the trans-theoretical model (39).

We conducted seven focus groups (*N* = 51) to explore barriers and facilitators of CRCS among Puerto Ricans living on the island. Inclusion criteria for focus group participants included: being a patient at a FQHCs, between the ages of 50–75, not being up to date with CRCS, and no previous diagnosis of CRC. We employed an adapted interview guide (13) that included questions regarding knowledge, attitudes, and beliefs about CRC and CRCS; as well as screening tests barriers and benefits. We also asked questions to clarify the process of obtaining a CRCS, information needs, and preferences for educational material. We transcribed the focus group recordings and used ATLAS.ti (version 7.5.10) for analysis. Members from the research team reviewed the transcripts (primary documents) independently and used constant comparative method (40) to identify themes and emerging topics. Two coders, one in PR (CV) and one in Houston (NFE), conducted an open coding identifying concepts and ideas using inductive methodology. The team reexamined the data according to differences and similarities, creating annotations in the form of memos. The resulting codes were classified into categories and subcategories (i.e., topics that were significant to respondents and are more generic concepts). Next, we continued with an axial coding that allowed us to clarify the relations between the different categories and subcategories. The team held weekly meetings to discuss coding and themes that had emerged. In most cases, coders identified the same themes. Any discrepancies in coding were resolved through team discussion. To assist in data reporting, we created categories of personal determinants and environmental factors that influence CRCS among Puerto Ricans living on the island (see Table 2).

**Table 2:** Personal determinants and environmental factors influencing colorectal cancer screening (CRCS) among Puerto Ricans.

Categories	Subcategories
Personal determinants influencing CRCS	Knowledge about CRC/CRCS Misconceptions about CRC/CRCS Low perceived risk for getting CRC
	Attitudes:
	Machismo Fatalism Procrastination
	Affective factors:
	Fear (concerning the colonoscopy procedure) Embarrassment (concerning the colonoscopy procedure) Fear (of the results of the test)
	Perceived structural barriers:
	Lack of time Transportation problems

Environmental factors influencing CRCS (interpersonal)	Lack of provider recommendation

#### Personal Determinants and Environmental Factors from Literature Review and Focus Groups

Based on the review of the empirical and theoretical literature we found that the following personal determinates influenced CRCS: low knowledge, perceived social norms, fear of finding CRC negative attitudes toward CRCS, perceived barriers, and low perceived risk (6, 8, 11–17). At the environmental level, substantial evidence from the literature pointed to lack of provider recommendation as a key environmental factor negatively influencing CRCS (6–10).

Results from the focus groups indicated that the following personal determinants influenced CRCS: lack of knowledge and misconceptions about CRC and CRCS; low risk perception about getting CRC; attitudes such as machismo, fatalism and procrastination; feelings of fear and embarrassment concerning the colonoscopy procedure, fear of test results, and perceived barriers such as lack of time and transportation problems. As in the literature, lack of provider recommendation was the primary environmental factor that emerged in the focus group findings. There was a high level of consistency in personal determinates and environmental factors identified through the literature review and focus groups.

#### Preferences Regarding Educational Intervention Components

When asked about the type of information they would like to receive, focus group participants indicated they would like to know more about CRC and CRCS tests, type of coverage government issued health insurance provided for CRCS, and where they could undergo testing. Participants said that it would be both helpful and important if health-care providers informed them about these issues. Participant preferences for educational materials included videos or printed materials with attractive images containing simple vocabulary.

### IM Step 2: Program Objectives

Based on the needs assessment, we defined the overall behavioral outcome: “Puerto Ricans ages 50 and older adhere to CRCS guidelines.” Once the overall behavioral outcome was established, we formulated performance objectives (i.e., what participants need to do to complete CRCS) (see Table 3). We then identified determinants of the positive behavioral outcome (i.e., why participants would complete de CRCS) rather than lack of CRCS (as in step 1). We examined determinants derived from step 1 and re-reviewed the literature and focus group findings to identify factors that would positively impact CRCS. These included knowledge, perceived risk, decisional balance, outcome expectations, self-efficacy/skills, perceived norms, and attitudes. According to IM step 2, we then created a matrix of change objectives by placing performance objectives in the left column and determinants across the top of the matrix. Then, for each determinant and the corresponding performance objective we asked: what has to change in relation to the determinant so that our population of interest can achieve the desired performance objective? These change objectives were recorded in the cells of the matrix (see Table 4).

**Table 3:** Behavioral outcome with associated performance objectives.

Behavioral outcome
Puerto Ricans ages 50 and older adhere to CRCS guidelines
**Performance objectives**
Make an appointment with the provider Discuss CRC and CRCS with the provider Request FIT/FOBT or obtain a referral for colonoscopy Identify location to get screened Make an appointment to get screened Arrange transportation Seek social support Get screened for FIT/FOBT Get screened for colonoscopy if recommended Record and keep appointment to the discuss the results with the provider

*FIT, fecal immunochemical test; CRCS, colorectal cancer screening; CRC, colorectal cancer; FOBT, fecal occult blood test*.

**Table 4:** Sample cells from matrix of change objectives.

Performance objectives	Overall behavioral outcome: “Puerto Ricans ages 50 and older adhere to CRCS guidelines”						
	Determinants						
	Knowledge	Perceived susceptibility (risk)	Decisional balance (pros and cons)	Outcome expectations	Self-efficacy/skills	Perceived social norms (subjective norms)	attitudes
PO8. Get screened FOBT/FIT (1) Pick up test from the lab or accept test from PCP (2) Read instructions (3) Complete test (4) Return test to lab	K8a. States that FOBT/FIT is recommended to be done annually for people over 50-year old K8b. Describes the steps to complete a FOBT or FIT K8c. Lists place where test should be returned	PR8. Perceives that he/she is at risk of getting CRC	DB8a. States the advantages of doing FOBT/FIT annually DB8b. Believes there are more benefits to CRCS than barriers DB8c. Describes ability to overcome barriers to doing FOBT/FIT annually	OE8a. Expects that if he/she gets CRCS they will reduce the risk of CRC or detect it early enough to be cured OE8b. Expects that going to pick up the test will result in getting one and getting tested OE8c. Expects that returning the test will result in quick information about outcome	SE8a. Expresses confidence in ability to pick up the test SE8b. Expresses confidence in ability to complete all steps SE8c. Expresses confidence in the ability to return the test	SN8a. Believes that other adults like them pick up the FOBT/FIT annually SN8b. Believes that other adults like them complete and return the test	ATT8a. Believes that CRCS is important ATT8b. Believes that early detected cancer can be cured ATT8c. Describes the importance of preventive behaviors such as screening to take care of one’s own health

PO9. Get screened for colonoscopy Identify someone to go with you	K9a. States that colonoscopy is recommended to be done every 10 years for people over 50-year old K9b. Describes the steps to complete a colonoscopy including preparations K9c. Lists place where to go for colonoscopy K9d. Describes need to get a referral from doctor		DB9a. States the advantages of doing colonoscopy DB9b. Believes there are more benefits to CRCS and colonoscopy specifically than barriers DB9c. Describes ability to overcome barriers to doing colonoscopy	OE9a. Expects that if he/she gets a colonoscopy they will reduce the risk of CRC or detect it early enough to be cured OE9b. Expects that getting a colonoscopy will allow them to wait 10 years before having to get another one (if negative)	SE9a. Expresses confidence in ability to identify someone to go with him/her to appointment SE9b. Expresses confidence in ability to conduct preparations SE9c. Expresses confidence in ability to complete colonoscopy	SN9a. Believes that other adults like them identify person to accompany them to the appt SN9b. Believes that other adults like them get colonoscopy SN9c. Believes that other adults like them complete preparations correctly	ATT9a. Believes that colonoscopy is useful and important because it can identify cancer and polyps (pre-cancer) ATT9b. Believes that correctly completing prep contributes to a more accurate test ATT9c. Believes that the test results will give them peace of mind ATT9d. Believes that even if embarrassed the test is worth it ATT9e. Believes that cancer if detected early can be cured

*CRC, colorectal cancer; CRCS, colorectal cancer screening; FIT, fecal immunochemical test; FOBT, fecal occult blood test*.

### IM Step 3: Program Design

We then identified theoretical change methods that are known to influence the determinants identified and conducted a literature review to help identify these methods as well as the practical applications or strategies to operationalize these. The intent of this step was to create strategies, materials, and messages that would address specific change objectives. We selected two overarching methods: entertainment education (38, 41, 42) and behavioral journalism (43). Entertainment education employs formats based on entertainment to introduce educational messages. In behavioral journalism, real-life role models who are identified as peers of the population of interest (with the same language and similar cultural and social norms) communicate the message (44, 45). These overarching methods also include other change methods including modeling, reinforcement, persuasion (Social Cognitive Theory), tailoring (Trans-Theoretical Model), anticipatory regret (Theory of Plan Behavior), consciousness raising (Health Believe Model), and providing cues to action (Theories of Information Processing). Strategies identified to operationalize these methods included testimonials and role-model stories about people talking with their provider about CRCS tests and overcoming barriers. We designed messages that include prompts for thinking about what might happen if they do not get screened and the regret that would accompany the decision.

Despite the importance of the organizational and provider level factors influencing CRCS identified during the needs assessment phase and our desire to create a multilevel intervention to address these factors, resources and project scope limited the ability of the team to do so. Nevertheless, since provider recommendation is an important and necessary component of any CRCS intervention, we decided to intervene using the patient activation method. This method is strongly associated with self-reported quality of care, a better doctor–patient communication, and increase CRCS rates (20, 46).

### IM Step 4: Creating Educational Components

In this step, we designed, produced and pretested the educational materials guided by the matrix of change objectives, methods, and strategies previously described. We reviewed the information obtained from the focus group analysis. Keeping in mind participants’ preferences about informational needs and educational materials format, and guided by the change objectives from the matrices developed in step 2, we created a series of drafts that conveyed messages and content. We then modified drafts according to the format and type of educational material that would be presented. All developed materials were produced in Spanish and designed to be culturally relevant and appropriate for Puerto Ricans and individuals with low or no literacy skills.

The educational program consists of four components: a Tailored Interactive Multimedia Intervention (TIMI), printed materials (newsletter, infographics, and action plan), a provider prompt, and a patient reminder support call. The TIMI was created based on entertainment education criteria (41, 42) in collaboration with media professionals and was designed to be delivered on tablet computers. The TIMI consists of a video with tailored scripted scenes and testimonials; narrations, animations and interactivity. During development, we held two script readings, one with professionals and collaborators and a second one with community members to assess language suitability and cultural acceptability of the scenes, narrations, and testimonials. The main recommendations were to clarify and simplify medical language about CRCS and expand the conversation that occurs in the physician–patient scenes about CRC and CRCS. We modified scenes and the script based on recommendations from these activities.

To develop the newsletter, we followed behavioral journalism techniques (47). We conducted 10 in-depth interviews with participants who had completed CRCS and had characteristics similar to those of our target population. We conducted journalistic style interviews that included specific questions related to key performance objectives and determinants so that stories would reflect the most relevant information needed. We included both individuals who were up to date with screenings, or who had survived CRC because completion of the CRCS tests. During the interviews, we used open-ended questions to obtain quotes from participants to be incorporated into program materials. During analysis of these interviews, we selected keywords and expressions regarding how participants overcame CRCS barriers and asked about how they felt about the outcomes of screening. We used these to create stories about the benefits of CRCS and about how these individuals had overcome barriers to receive screening and protect their health.

We also developed an infographic and an action plan. These included images and messages with information about CRC and CRCS, steps to follow to complete CRCS tests, and mini-testimonials from people who completed CRCS.

As mentioned earlier, we created a provider prompt based on the patient activation method. This method enables patients to assume an active role in their health care (46, 48). Specifically, we used a patient mediated approach in which the patient gives the provider printed information about CRCS with questions or concerns they may have to prompt discussion; the prompt, a short summary of patient need for CRCS was printed from the TIMI following an interactive session. This also serves as a cue to action for the provider. We designed the prompt to be tailored to knowledge, attitudes, and barriers about CRCS that the participants reported during use of the TIMI.

We conducted focus groups (*N* = 19) and administrated a survey to test the appeal, acceptability, perceived relevance, cultural appropriateness, and motivation to obtain a CRCS of the TIMI and printed materials. Overall, participants found the educational components to be both attractive and culturally sensitive. Most participants indicated that their knowledge regarding CRC and CRCS increased and that they felt motivated to complete screening after viewing and reading the different materials. The only changes participants suggested were to reduce the length of the text in the printed materials and to incorporate information about CRC prevention. We used this information to refine the messages and the quality of the components.

## Discussion

In this article, we describe the development of an intervention to increase CRCS using IM. We described the first four steps used in the development process. The last two steps of IM, planning for implementation and evaluation, are currently underway and will be described in a subsequent paper. By using the process of IM, we ensured the systematic incorporation of theory and evidence from the literature and new data from the community participation to address the personal determinants and environmental factors using an ecological perspective. IM also guided the selection of the most appropriate methods and practical strategies, as well as the design and creation of the educational components of the program.

We found that entertainment education and behavioral journalism were two overarching effective methods relevant for addressing the identified personal determinants and environmental factors. These methods that are aimed at changing social norms attitudes, and self-efficacy (21) are particularly effective in reaching audiences that may have low literacy, or who are initially resistant or unwilling to process the message (43, 49). As strategies (practical applications) to operationalize these methods, we included testimonials, role-model stories and patient mediated provider prompts aimed at showing how to overcome barriers to complete the screening tests. The *Salud por la vida* program we developed using IM is a multicomponent intervention. Shokar et al. and Sabatino et al. both conclude that the use of multicomponent interventions is more effective for increasing CRCS testing uptake than the individual components by themselves (16, 50, 51). Likewise, a study with health promoters showed that participants who received a one-on-one educational intervention in combination with patient reminders for FOBT were more likely to get tested than those who just received the patient reminder (31). Another study with lay health workers reinforced the importance of testing small media approaches in combination with one-on-one educational interventions (12). The development of this intervention is expected to help to fill this gap by using multiple strategies to increase CRCS.

Using IM to design *Salud por la Vida* provided an organized, structured, and systematic approach to program development that helped guide (a) the identification of relevant factors influencing CRCS, (b) how and when to use theory and empirical evidence to make decisions about change methods, practical applications, materials, and messages, and (c) how to best engage the community in the planning process to ensure regular communication and feedback.

## Limitations

Due to the qualitative nature of some of the activities of this study such as focus groups, results are not generalizable to all populations 50 years of age and older in PR. Nevertheless, participants were selected to represent the target population for the intervention, therefore, the identified determinants of CRCS are likely those most relevant. In addition, most of those who participated in the behavioral journalism interviews were of higher socioeconomic status and had a better access to private health insurance than the island’s general population. Nevertheless, participants identified similar barriers to those documented for lower socioeconomic status. In addition, those with private health insurance recognized that their situation was different to that of the general population and related the experiences of friends and family members who do not have private health insurance.

## Ethics Statement

The study, its components, and protocol were approved by the Institutional Review Boards of the UPR-MSC and the University of Texas Health Science Center at Houston.

## Author Contributions

MF, VC-L, LS, and SV: program conceptualization. MF, YS-M, VC-L, NF-E, CV, and AA: development of the program. YS-M and MF: manuscript development and primary writers.

## Conflict of Interest Statement

The authors declare that the research was conducted in the absence of any commercial or financial relationships that could be construed as a potential conflict of interest.
